# Functional morphology of the blood-brain barrier in health and disease

**DOI:** 10.1007/s00401-018-1815-1

**Published:** 2018-02-06

**Authors:** Stefan Liebner, Rick M. Dijkhuizen, Yvonne Reiss, Karl H. Plate, Dritan Agalliu, Gabriela Constantin

**Affiliations:** 1 Institute of Neurology, Goethe University Clinic, Frankfurt am Main, Germany; 2 Excellence Cluster Cardio-Pulmonary Systems (ECCPS), Partner site Frankfurt, Frankfurt am Main, Germany; 3 German Center for Cardiovascular Research (DZHK), Partner site Frankfurt/Mainz, Frankfurt am Main, Germany; 4 Center for Image Sciences, University Medical Center Utrecht and Utrecht University, Utrecht, The Netherlands; 5 German Cancer Consortium (DKTK), Partner Site Frankfurt/Mainz, Frankfurt am Main, Germany; 6 German Cancer Research Center (DKFZ), Heidelberg, Germany; 7 Departments of Neurology, Columbia University Medical Center, New York, NY 10032, USA; 8 Departments of Pathology and Cell Biology, Columbia University Medical Center, New York, NY 10032, USA; 9 Departments of Pharmacology, Columbia University Medical Center, New York, NY 10032, USA; 10 Departments of Columbia Translational Neuroscience Initiative, Columbia University Medical Center, New York, NY 10032, USA; 11 Department of Medicine, Section of General Pathology, University of Verona, Verona, Italy

**Keywords:** Blood–brain barrier, Steady state, Stroke, Brain tumour, Neuroinflammation, Alzheimer’s disease

## Abstract

The adult quiescent blood–brain barrier (BBB), a structure organised by endothelial cells through interactions with pericytes, astrocytes, neurons and microglia in the neurovascular unit, is highly regulated but fragile at the same time. In the past decade, there has been considerable progress in understanding not only the molecular pathways involved in BBB development, but also BBB breakdown in neurological diseases. Specifically, the Wnt/β-catenin, retinoic acid and sonic hedgehog pathways moved into the focus of BBB research. Moreover, angiopoietin/Tie2 signalling that is linked to angiogenic processes has gained attention in the BBB field. Blood vessels play an essential role in initiation and progression of many diseases, including inflammation outside the central nervous system (CNS). Therefore, the potential influence of CNS blood vessels in neurological diseases associated with BBB alterations or neuroinflammation has become a major focus of current research to understand their contribution to pathogenesis. Moreover, the BBB remains a major obstacle to pharmaceutical intervention in the CNS. The complications may either be expressed by inadequate therapeutic delivery like in brain tumours, or by poor delivery of the drug across the BBB and ineffective bioavailability. In this review, we initially describe the cellular and molecular components that contribute to the steady state of the healthy BBB. We then discuss BBB alterations in ischaemic stroke, primary and metastatic brain tumour, chronic inflammation and Alzheimer’s disease. Throughout the review, we highlight common mechanisms of BBB abnormalities among these diseases, in particular the contribution of neuroinflammation to BBB dysfunction and disease progression, and emphasise unique aspects of BBB alteration in certain diseases such as brain tumours. Moreover, this review highlights novel strategies to monitor BBB function by non-invasive imaging techniques focussing on ischaemic stroke, as well as novel ways to modulate BBB permeability and function to promote treatment of brain tumours, inflammation and Alzheimer’s disease. In conclusion, a deep understanding of signals that maintain the healthy BBB and promote fluctuations in BBB permeability in disease states will be key to elucidate disease mechanisms and to identify potential targets for diagnostics and therapeutic modulation of the BBB.

## The BBB under steady state conditions

### Introduction

**Fig. 1:**
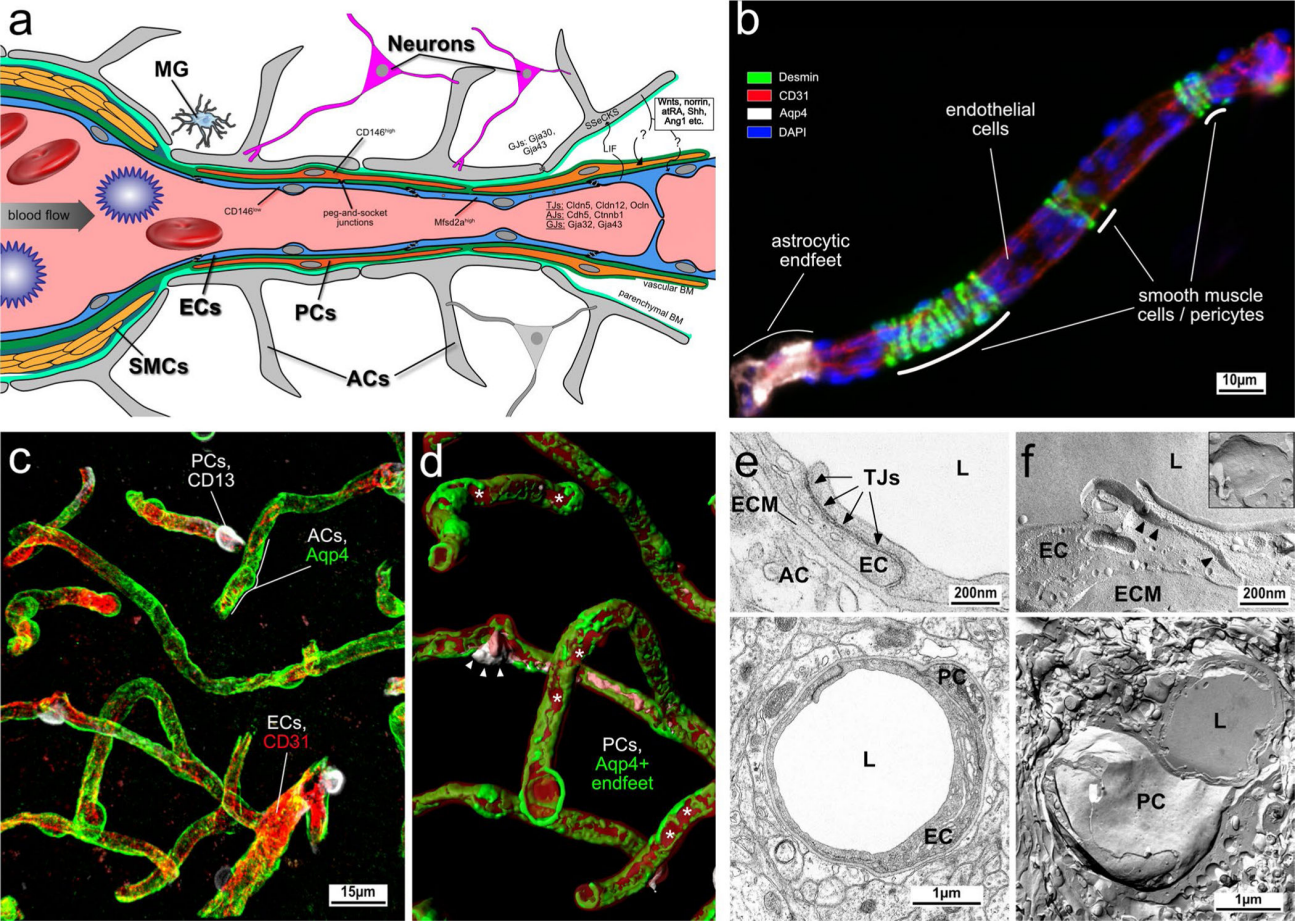
Morphological and functional characteristics of the healthy NVU and BBB. **a** Scheme of brain vessels ranging from an arteriole, via a capillary to a venule, representing the cellular and molecular composition of the NVU. **b** Isolated mouse cortical micro vessel stained for CD31, desmin, Aqp4 and DAPI. Due to the mechanical and chemical isolation process, only remnants of PCs and AC end-feet are detectable at the CD31 + vessel. **c** Mouse cortical vessels stained in vibratome sections for CD31, CD13 and Aqp4, demonstrating almost complete coverage of vessels by AC endfeet. PCs indicated by arrowheads are located on microvessels and cling around them with their cellular processes. **d** Magnified ROI of C and 3D rendered, demonstrating partially incomplete astrocytic end-feet coverage of endothelial cells (asterisks) as well as PC cell body (arrowheads) and processes (arrows). **e** Transmission electron microscopy showing a cortical capillary with ECs, PCs and ACs with interendothelial junctions (lower panel). Higher magnification of interendothelial junctions (upper panel) (kindly provided by Jadranca Macas, Institute of Neurology, Goethe University Clinic Frankfurt, Germany). **f** Freeze fracture preparation of a cortical capillary with an attached pericyte (lower panel). Freeze fracture of interendothelial junction (upper panel), arrowheads show junctional strands. Inset shows junction strands in higher magnification (kindly provided by Hartwig Wolburg, Institute of Pathology, University Clinic Tübingen, Germany). ECs, endothelial cells; PCs, pericytes; SMCs, smooth muscle cells; ACs, astrocytes; MG, microglia

The blood–brain barrier (BBB) is instrumental in establishing and maintaining the microenvironment of the central nervous system (CNS) that permits proper neuronal function. Since the establishment of the BBB concept by Paul Ehrlich, Edwin Goldmann and Lena Stern about a century ago, the morphological, molecular and physiological properties of the BBB have been extensively studied. Although the pioneering work of Reese and Karnovsksi as well as Brightman and Reese in the late 1960s identified that CNS endothelial cells (ECs) are the site of the BBB proper, it has become apparent that ECs require contacts with various CNS cell types to establish BBB characteristics (for review see [27]). The close contact of ECs with pericytes (PCs) via the “peg-and-socket” junctions within a common basal lamina and the ensheathment of brain capillaries by astrocytic endfeet are crucial for establishing and maintaining the BBB [24]. Moreover, interneurons and perivascular microglia also make contacts with ECs, pericytes and astrocytes to establish a neuro-vascular unit (NVU) (Fig. 1a), a term that reflects both morphological relationships as well as molecular interactions between these various cell types.

However, not all CNS ECs share the same morphological, molecular and physiological characteristics. This regional vascular heterogeneity is probably best described for structures like the choroid plexus or the circumventricular organs (CVOs) whose blood vessels lack a BBB to allow secretion of the cerebrospinal fluid and neurosecretory/-sensory function, respectively [113]. In recent years, it has become more evident that microvessels do not share identical barrier features even within CNS regions that have a proper BBB [134]. Multiple genetic studies with global or conditional transgenic mouse lines have identified that Wnt7a/7b ligands and the Wnt/β-catenin pathway are major molecular drivers of angiogenesis and barriergenesis in the brain [35] (Fig. 1a). The specificity of Wnt ligand binding and downstream pathway activation in the brain depend on specific co-receptors in ECs such as *G-protein coupled receptor 124* (Gpr124) [147] and *reversion-inducing cysteine-rich protein with Kazal motifs* (RECK) [25, 129]. However, the molecular players that activate the β-catenin pathway in the retina and cerebellum are quite distinct. Genetic ablation of the Norrin disease protein (Ndp) ligand, Wnt receptor Frizzled 4 (Fzd4), Lrp5 and the co-receptor Tetraspanin-12 (Tspan-12) result in defective angiogenesis and barrier disruption in both retinal and cerebellar vessels [25, 148]. These studies demonstrate regional differences in both molecular and ultimately physiological aspects of the BBB within the CNS. Blood vessels in the retina, which form a blood-retina barrier (BRB), have also distinct PC attachment and astrocyte coverage from their brain counterparts [17]. Investigation of these regional differences is a major focus in current BBB research in order to identify relevant physiological function of the BBB within specific CNS regions and to develop potential drug targets for neurological pathologies like ischaemic stroke, tumour, neuroinflammation, Alzheimer’s diseases affecting certain CNS regions.

Below, we describe and discuss the BBB under steady-state condition and selected pathologies of the brain, focusing on ischaemic stroke, brain tumours, neuroinflammation and Alzheimer’s disease to illustrate the cellular and molecular mechanisms affecting BBB function in these diseases and identify potential diagnostic and therapeutic progress ultimately affecting patient survival.

## Endothelial cells under steady-state conditions

Brain ECs are characterised by elaborate tight junctions (TJs) that are formed primarily by the endothelial-specific claudin family member claudin-5 (Cldn5) and occludin (Ocln) (Fig. 1a, e, f). These proteins are linked to the cytoskeleton by members of the zonula occludens family (ZO-1, −2, −3) (Fig. 1a). Cldn5 plays a crucial role for TJ formation and BBB paracellular function, since embryonic ablation of Cldn5 in mice leads to early postnatal brain oedema and lethality (reviewed in [35]). Moreover, Cldn5 is regulated in vivo by the Wnt/β-catenin pathway in an opposing manner to that of the permeability associated protein plasmalemma vesicle-associated protein (Plvap; also known as Meca-32) (reviewed in [35]). However, Cldn5 regulation by β-catenin appears to be highly context dependent. Corada and colleagues have demonstrated that Cldn5 is inhibited, rather than activated, by β-catenin when the transcription factor FOXO-1 that is normally induced by vascular endothelial growth factor (VEGF) signalling is active in ECs. Thus, activation of other signalling pathways (e.g. VEGF) may counteract the ability of β-catenin signalling to regulate transcriptional targets important for BBB function (reviewed in [35]). Cldn5 overexpression does not lead to high resistant TJs in ECs in vitro, suggesting that other TJ proteins like Cldn3 and −12, play an important role to regulate the paracellular barrier of brain ECs [35]. However, the contribution to BBB function of these additional TJ-associated proteins and their structural and molecular integration is still under debate. Several studies have documented expression and regulation of Cldn3 in brain ECs in vitro and in vivo [126]; however, there is no direct evidence that Cldn3 is required for BBB function. Recently, Cldn3 has been shown to be instrumental in maintaining blood-cerebrospinal fluid barrier (BCSF) in epithelial cells under chronic inflammatory conditions (reviewed in [126]). Further studies using endothelial-specific deletion of Cldn3 or other members of the claudin family are needed to determine their contribution to BBB paracellular permeability. Members of the IgG superfamily such as EC adhesion molecule (ESAM), junction adhesion molecules (JAM1–3) and nectins are also associated with TJs, and have been shown to regulate TJs function as well as transmigration of inflammatory cells, particularly leukocytes, across the endothelial barrier [33]. However, their detailed function at the BBB is not fully established. Finally, a class of TJ-associated proteins such as tricellulin [53] and lipolysis-stimulated lipoprotein receptor (LSR) [79] are localised at the point of connection between three cells (so-called tricellular junctions), which are weak junctional points between cells. Tricellulins play an essential role to stabilise these specialised junctions in epithelial cells; however, their role at the BBB is unknown. Genetic deletion of LSR results in hampered formation of a proper paracellular endothelial barrier during development and increased leakiness to small molecules [114], suggesting that these specialised tricellular junctions may play an important role at the BBB.

Although TJs regulate the paracellular endothelial barrier properties, adherens junctions (AJs), formed by homophilic interactions between cadherins, are considered a prerequisite for the establishment of TJs (for review, see [29]). Vascular endothelial (VE)-cadherin (Cdh5, CD144) is the major cadherin family member in ECs. N-cadherin is the second most expressed cadherin family member in ECs and mediates interaction between ECs and PCs via the peg-and-socket junctions. Although AJs are present in all ECs, their molecular composition and consequently their functional properties differ between distinct types of vessels [29]. Both VE- and N-cadherin function also as adhesion receptors and are involved in downstream signalling via a complex of proteins bound to their cytoplasmic tails. Beside its function as a co-transcription factor in the canonical Wnt pathway mentioned above, β-catenin and its close homologue plakoglobin/γ-catenin promote VE-cadherin anchorage to actin microfilaments.

A second unique feature of the BBB is the presence of various facilitated and active transport mechanisms in ECs that confer nourishment of the CNS as well as transport of waste products out of the CNS. The best described transporter is the glucose transporter isoform 1 (Glut-1, Slc2a1) that becomes highly expressed upon BBB formation and has been shown to be regulated by the Wnt/β-catenin pathway [35]. Among the plethora of transporters expressed in brain ECs, the thyroxine transporter, belonging to the solute carrier organic anion transporter family member (Slco1c1), has been exploited to drive brain endothelial-specific expression of the Cre recombinase as a valuable tool to investigate BBB-specific gene function [102]. BBB ECs also express members of the ATP-binding cassette family of transporters, like Abcg1a1 (Mdr, Pgp), Abcg2 (Bcrp), Abcc1 (Mrp1) and others. The most intensively studied ABC transporter certainly is Pgp, that is a BBB identity gene, and has been implicated in barrier homeostasis and in the progression of various diseases like Alzheimer’s disease and others (for review see [83]).

A third feature of the BBB is the presence of a small number of caveolae and reduced transcellular transport [27, 35]. Recently, a transporter of the major facilitator superfamily domain-containing (Mfsd) family, specifically Mfsd2a has been characterised as a transporter for the essential omega-3 fatty acid docosahexaenoic acid (DHA) in brain ECs that contribute to the regulation of vesicular traffic in CNS BBB/BRB endothelia [12]. Interestingly, the expression of Mfsd2a becomes upregulated in CNS ECs along with the formation of BBB characteristics. Systemic gene ablation of Mfsd2a in mice resulted in BBB leakiness due to significantly increased vesicular traffic in ECs. Mfsd2a is located upstream of cave- olin-1 (Cav-1) and regulates the membrane lipid composition of CNS ECs that in turn influences the formation of caveolar-rich domains within the membrane [26]. Cav-1 is a well-known marker of caveolae in endothelial cells and plays an important role in endothelial physiology and pathology, regulating functions like signal transduction, endocytosis, transcytosis, and molecular transport. Specifically, Cav-1 regulated the angiogenic response by influencing VEGF receptor 2 (VEGFR2) phosphorylation and internalisation [145]. In the context of junctional opening, Cav-1 was shown to interact with β-catenin and to thereby weaken the VE-cadherin-based AJs [64]. At the BBB, Cav-1 was described to relate to diminished TJ proteins expression upon BBB disruption. Cav-1 cooperates with several pathways, serving as a signalling platform. Genetic ablation experiments of Cav-1 revealed a major function for mediating transcellular but not paracellular permeability in BBB ECs.

## Pericytes under steady-state conditions

Genetic manipulations in mice that change the coverage of blood vessels by PCs have shown that PCs play a crucial contribution to BBB integrity, in particular via the regulation of the transcellular barrier [124]. Genetic ablation of Mfsd2a and PC vessel coverage do not affect TJs integrity and suggest that the paracellular BBB pathway is regulated independently from the transcellular pathway. Recent studies from Chow and Gu have shown that BRB maturation is controlled by down regulation of vesicular transport (transcellular permeability), rather than by improvement in the junctional seal between ECs (paracellular barrier) [26], supporting the independent regulation of the two pathways that control BBB/BRB function. PCs are the cells of the NVU most firmly attached to the endothelium, and as cellular “relatives” of smooth muscle cells they may regulate vessel diameter, thereby controlling physiological parameters of blood supply [124], although these findings are controversial. One way in which PCs communicate with ECs are via the “peg-and-socket” junctions which are formed by N-cadherin and connexin-43 hemichannels, and may allow transport of nutrients and signalling metabolites [124]. However, it is not clear how PCs communicate with ECs and which are the molecular regulators involved in this mutual interaction. Chen et al. have recently shown that the single cell adhesion receptor CD146 (syn.: melanoma cell adhesion molecule (MCAM) or cell surface glycoprotein MUC18) functions as a co-receptor for PDGFR-β on PCs to regulate PC-EC interactions [23]. Interestingly, CD146 is differentially expressed during BBB maturation, showing initial expression on ECs that are devoid of PCs, followed by its endothelial downregulation upon PC recruitment and BBB maturation. The latter happens to be promoted by TGFβ−1 secretion upon PCs attachment. Although several studies have shown that PCs are essential for BBB formation and maintenance, astrocytes (ACs) are also considered as a major source for barrier inducing and maintaining signals [24]. It is conceivable that CNS-intrinsic cues, including those released from astrocytes, might be required to complete and/or supplement the barrier-promoting signals derived from PCs.

## Astrocytes under steady-state conditions

Astrocytes (ACs) are the most numerous cell type in the vertebrate CNS and their specialised end-feet cover nearly the entire surface of CNS microvessels. ACs develop at late gestation stages (mouse ~ E17.5) from radial glia (RG) and common neural precursor cells (NPC), suggesting that early BBB-inducing events are likely not mediated by ACs [24]. However, ACs are in close association with brain microvessels and various AC proteins like aquaporin-4 (Aqp4) and the potassium channel Kir4.1 are localised at the end foot membrane to regulate water homeostasis at the NVU (Fig. 1b, c, d) (for review see [35]). Moreover, Aqp4 has been implicated in the flow of the interstitial fluid (ISF) as part of the so-called “glymphatic system” (discussed in detail in the manuscript by Abbott et al. in this cluster). ACs improve endothelial barrier function either in co-culture models or by the administration of conditioned medium to the EC single cultures in vitro [24]. Moreover, postnatally ACs are the major source of Wnts and norrin that were identified as crucial factors of BBB maintenance [148]. Additionally, ACs secrete sonic hedgehog (Shh), retinoic acid (RA) and angiopoietin-1 (Ang-1), all key factors that induce or support barrier properties of brain ECs [5, 43]. AC–EC interactions are essential not only for the establishment and maintenance of the BBB, but also for astrocyte differentiation. ECs secrete factors like leukaemia inhibitory factor 1 (LIF1) that supports ACs differentiation. This in turn induces expression of Src-supressed C-Kinase substrate (SSeCKS), leading to the astrocytic secretion of Ang-1 that stabilises vessels through Tie2/TEK binding on ECs (reviewed in [35]). It is not entirely clear which AC-derived factors including interleukin-6 (IL-6), glial cell line-derived neurotrophic factor (GDNF) or fibroblast growth factor 2 (bFGF) are crucial for BBB maturation and maintenance. Hence more work needs to be done to unravel the molecular crosstalk not only between ECs and ACs, but also between PCs and ACs.

## Neurons, microglia and extracellular matrix components under steady-state conditions

Since ACs develop during late embryonic and postnatal stages to support BBB maintenance, but not induction, neural precursor and early neurons were shown to be the main source of Wnt7a and Wnt7b that promote brain vascularization and the formation of the BBB (for review see [35]). Once the NVU is fully established and the BBB is mature, neurons do not or infrequently have direct contact with brain ECs, suggesting that ACs and PCs may take over for BBB maintenance. However, both PCs and ACs directly signal to neurons to modulate synaptic strength and also convey signals from neurons to the vasculature, ultimately leading to arteriolar dilation and an increase in local blood flow, thereby influencing BBB physiology [10]. Microglia are abundant resident immune cells in the CNS that display little turnover under physiological conditions. Due to their perivascular localisation they screen the brain parenchyma for blood-borne substances and potential inflammatory stimuli, providing a first line of defence in the CNS [120]. During brain angiogenesis, microglia and macrophages have been implicated in the regulation of vascular growth, by secretion of attractive as well as repellent signals including Wnts (for review see [35]). Once vascularisation is completed and the BBB is established, the role of the resident microglia for BBB maintenance is less clear, given the fact that postnatal ablation of microglia does not result in BBB impairment [120]. Hence the role of microglia under steady-state conditions at the BBB has to be investigated in more detail.

The extracellular matrix (ECM) components at the BBB are also crucial for its establishment and integrity [9]. ECs are embedded in a common basement membrane (BM) with PCs; both cell types contribute to secretion of components like collagen IV and fibronectin, as well as laminin 411 (α4β1γ1), laminin 511 (α5β1γ1) and laminin 211 (α2β1γ1). Astrocytes, however, secrete laminin 111 (α1β1γ1) and 211 (α2β1γ1), as well as nidogen, perlecan and agrin [9]. Agrin is considered to have important function in establishing and maintaining AC end foot polarisation and consequently Aqp4 clustering in orthogonal arrays of particles (OAPs). The main receptors of ECM components are dystroglycan as well as members of the integrin family of adhesion receptors. Genetic ablation experiments in mice have revealed that integrin αvβ8 leads to haemorrhages in the brain, likely by inhibiting TGFβ-mediated EC differentiation [9]. This finding also nicely illustrates that the ECM is a rich source for growth factors like latent TGFβ binding protein (Ltbp), VEGF, Wnts and other growth factors. To further unravel the specific role of ECM components at the BBB and their involvement in BBB maintenance, additional cell-specific and inducible loss- and gain-of-function approaches in mice need to be conducted.

## In vivo imaging of blood–brain barrier permeability after ischaemic stroke

Stroke, caused by interrupted blood flow to the brain, is among the leading causes of death and disability worldwide. Over the past two decades, treatment options for ischaemic stroke—the major stroke subtype—have substantially improved with the availability of clinically approved strategies to induce reperfusion of ischaemic brain tissue, i.e. intravenous pharmacological thrombolysis with recombinant tissue plasminogen activator (rt-PA) or endovascular mechanical thrombectomy. However, despite their potential to salvage affected brain tissue and to improve functional outcome after acute ischaemic stroke, these reperfusion therapies are hampered by a relatively short therapeutic timewindow of a few hours because of risk of bleeding.

Haemorrhagic transformation after ischaemic stroke is typically preceded by disruption of the blood–brain barrier (BBB). BBB leakage develops in the first hours after stroke and may contribute to progression of ischaemic tissue damage, secondary neuro-inflammation, vasogenic oedema formation and intracerebral bleeding [142]. Timely reperfusion may restore BBB permeability; however, late reperfusion can aggravate BBB injury, as a consequence of production of reactive oxygen species (ROS) and release of proteases [e.g. matrix metalloproteinases (MMPs)] from infiltrated leukocytes [56, 142]. This implies that the time and extent of BBB disruption are critical factors in the outcome of spontaneous or therapeutically induced reperfusion after stroke. Characterization of the BBB status can therefore provide valuable information on the condition and fate of ischaemic brain tissue, and may aid in treatment decision-making and monitoring following acute ischaemic stroke.

Over the years, several in vivo imaging methodologies have been developed to measure the integrity of the BBB. Magnetic resonance imaging (MRI), computed tomography (CT)/single-photon emission computed tomography (SPECT), or optical imaging, combined with intravenous injection of paramagnetic, radiolabelled or fluorescent markers, respectively, enable detection of contrast agent leakage across the BBB. The following sections will describe how different imaging approaches can inform on the degree, pattern, origin and consequences of BBB injury in clinical and experimental studies.

## MRI of BBB leakage after stroke

MRI of BBB leakage is determined from the extravasation and subsequent tissue accumulation of intravenously injected paramagnetic contrast agent, typically gadolinium (Gd)-based media, such as Gd-diethylenetriaminepentaacetic acid (Gd-DTPA). Parenchymal Gd-induced signal enhancement on T_1_-weighted MR images early after thrombolytic therapy has been shown to be predictive of subsequent symptomatic intracerebral haemorrhage in acute ischaemic stroke patients [49, 80]. In an embolic stroke model in rats, it was found that clear Gd-induced T_1_-weighted signal intensity enhancement, which preceded post-treatment bleeding, was already detectable before rt-PA treatment, particularly in regions with low CBF [32].

**Fig. 2:**
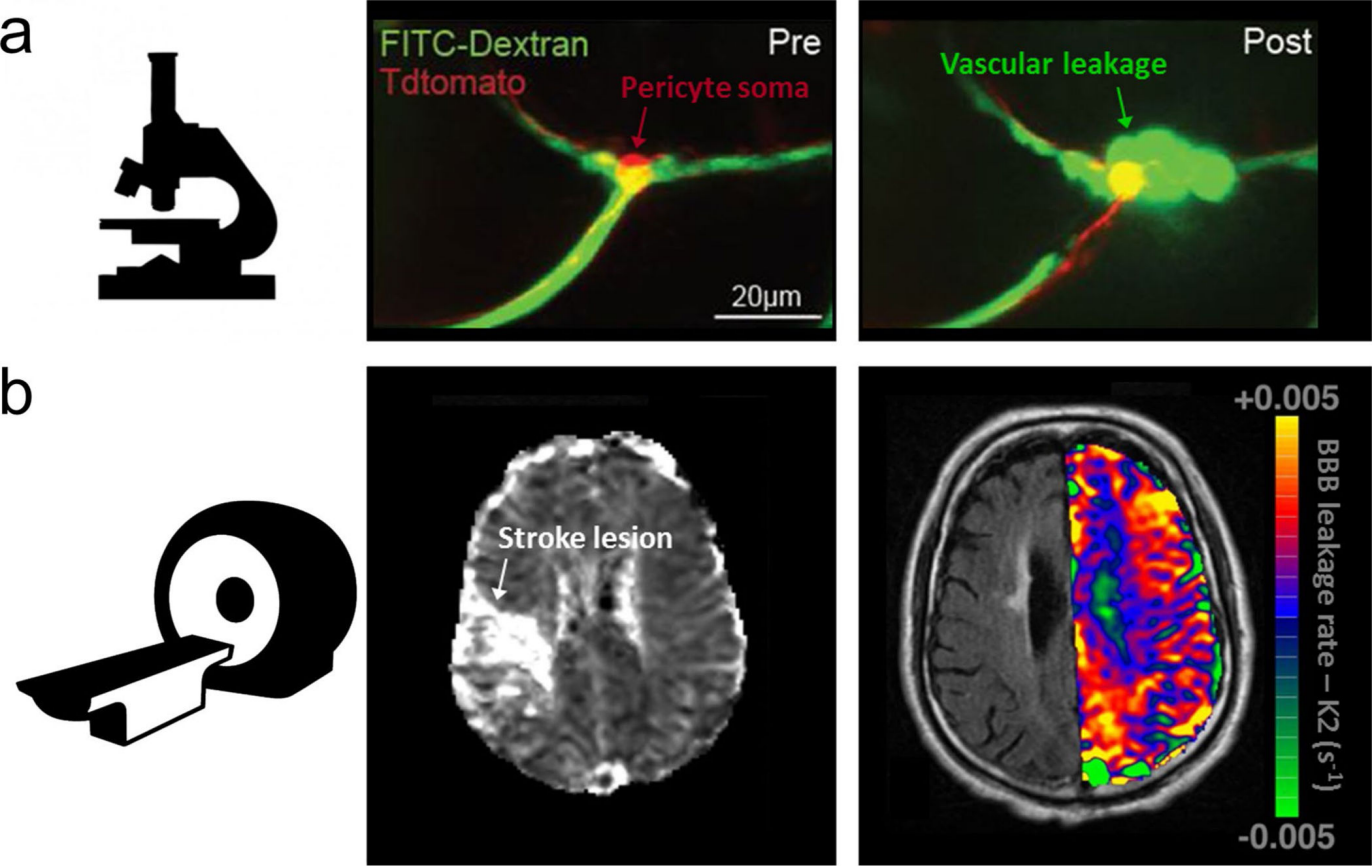
Imaging BBB permeability after stroke—from microscopic detection in mice to whole-brain assessment in patients. **a** Two-photon microscopic imaging, pre and post photothrombotic vessel occlusion, showing post-stroke capillary BBB leakage (green arrow) occurring particularly at pericyte somata (red arrow) in mouse brain (kindly provided by Robert Underly and Andy Shih, Medical University of South Carolina, Charleston, SC, USA) (for details see [127]). **b** MRI of increased BBB leakage rate (right image; colour coding) in contralesional white matter in a patient with an acute unilateral ischaemic stroke lesion (left image; hyperintensity on perfusion MRI-derived mean transit time map) (kindly provided by Ona Wu, Massa-chusetts General Hospital and Harvard Medical School, Boston, MA, USA) (Reprinted by Permission of SAGE Publications, Ltd.) (for details see [104])

While severe focal BBB leakage can lead to haemor-rhagic transformation after thrombolysis, mild diffuse BBB permeability may be reversible upon early reperfusion [112]. Small and transient increases in BBB permeability, detectable with dynamic contrast-enhanced (DCE) MRI (see also the next section on quantification of BBB permeability), may even occur outside the ischaemic territory during the first days after stroke [130]. Furthermore, MRI-based measurement of BBB leakage in contralesional white matter of acute ischaemic stroke patients has been found to be associated with elevated plasma MMP-2 levels and loss of microstructural white matter integrity (Fig. 2) [104]. Remote and widespread BBB leakage may reflect diffuse microvascular dysfunction, which could contribute to poor outcome after stroke. This indicates the potential of whole-brain imaging of BBB permeability as a complementary measure for the diagnosis of acute ischaemic stroke patients with possible microangiopathy. Interestingly, at later stages, MRI of BBB permeability may provide insights in post-stroke vascular remodelling or angiogenesis, possibly associated with functional recovery, as demonstrated in rodent studies [55].

Post-contrast MR images are usually acquired within 30 min after contrast agent injection, i.e. in the same scanning session. However, late gadolinium enhancement of cerebrospinal fluid space can be observed on follow-up fluid-attenuated inversion recovery (FLAIR) MR images of acute ischaemic stroke patients [66]. This phenomenon is strongly related to reperfusion and has, therefore, been termed “hyperintense acute reperfusion marker” (HARM). HARM has been recognised as a sign of early BBB breakdown and reperfusion injury, and its occurrence after thrombolysis has been associated with a higher incidence of post-procedural haemorrhages and worse neurological recovery [66].

## Quantification of BBB permeability

Detection of signal enhancement on post-contrast images is the most straightforward way to identify regions with a leaky BBB. In addition to this qualitative assessment, BBB permeability can be quantified with DCE or perfusion imaging protocols on MRI or CT scanners.

For DCE imaging, MR or CT images are serially acquired before, during and after injection of a low-molecular weight paramagnetic or iodinated contrast agent, respectively. The efflux rate of contrast agent from blood plasma into the tissue extravascular extracellular space, i.e. the volume transfer constant, K^trans^ (or blood-to-brain transfer constant, K_i_), or permeability-surface area product (PS), can be calculated from pharmacokinetic modelling of DCE signal intensity changes [117]. Common models are based on exchange between intra- and extravascular compartments. A popular analysis approach involves the Patlak plot model, a graphical analysis method that has been successfully validated in a rat stroke model [37], which appears particularly suitable for detection of subtle BBB permeability [48].

${\text{T}}_{2}^{*}$-weighted dynamic susceptibility contrast-enhanced (DSC) MRI or dynamic CT perfusion imaging are routinely used for measurement of haemodynamic indices, but also allow calculation of parameters, such as relative recirculation, that have been shown to be related to BBB integrity [139]. Although spatial resolution is usually lower than with DCE MRI, a major advantage of these rapid imaging methods is their short acquisition time (typically less than two minutes). In a multicentre retrospective study, permeability measures derived from DSC MRI in acute ischaemic stroke patients were found to provide an average haemorrhage prediction accuracy of more than 85% [107]. Similarly, increased PS values calculated from dynamic CT perfusion data have been shown to be predictive of haemorrhagic transformation in acute ischaemic stroke patients [71].

## Different contrast agents for BBB assessment

Diverse contrast agents with different dimensions and pharmacodynamic properties are available for assessment of BBB disruption. Although most of these agents are not approved for clinical use, they can be employed to experimentally investigate details and dynamics of BBB dysfunction in animal models. Application of contrast agents that differ in size enables assessment of varying BBB pore dimensions [146], while contrast agents with a long circulation life-time allow longitudinal monitoring of the evolution of BBB breakdown [32, 72]. For example, with MRI-detectable superparamagnetic long-circulating micron-sized iron oxide particles it was demonstrated that late delivery of rt-PA after embolic stroke in rats may rapidly incite extravasation of the circulating iron oxide particles, reflective of abrupt aggravation of microvascular barrier injury (see [32]). Build-up of iron oxide particles (or other microor nano-probes) in acute ischaemic tissue probably mostly results from passive diffusion through disrupted BBB [30]; however, intravenously injected iron oxide particles may also be taken up by (circulating) phagocytes, enabling MRI- based detection of inflammatory cells accumulating in or around ischaemic lesions [28]. This becomes particularly prominent at subacute stages after stroke when cellular infiltration is highest.

The availability of a variety of fluorescent vascular tracers also renders optical imaging methods applicable for in vivo studies on BBB dysfunction in animal models. In mice, time-domain near-infrared fluorescent (NIRF) imaging of optical tracers with different molecular weights has shown that the BBB is open to leakage of small as well as large molecular tracers at 24 h after 60-min middle cerebral artery occlusion (MCAo) [2]. Furthermore, in the same study bilateral and biphasic BBB disruption was observed with longitudinal NIRF of a small intravascular tracer during the first week after 30 min MCAo, which resolved after 14 days. The dynamics of BBB permeability after acute ischaemic stroke remain incompletely understood. Biphasic opening in relation to ischaemia–reperfusion injury has been frequently described [142], while there are also (MRI) reports of continuous BBB leakage from acute to subacute or chronic stages after (transient) cerebral ischaemia in rodents and human patients (for review see [80]). In a study that combined MRI and NIRF imaging in mice that underwent 60-min MCAo, co-injection of Gd-DTPA and bovine serum albumin (BSA)-conjugated indotricarbocya- nine revealed dissimilar extravasation patterns for the two contrast agents [60]. Gd-DTPA enhancement was detected at all time-points, whereas accumulation of the NIRF tracer was biphasically apparent at 8 and 16 h after reperfusion. These findings highlight the effect of contrast agent features, such as molecular weight and pharmacodynamics (BSA conjugates have relatively long plasma half-lives), on the leakage profile and time-course.

Most contrast agents are not approved for clinical use and even clinically approved contrast agents may have negative side effects. It is now well recognised that Gd-DTPA, which is primarily cleared by the kidneys, can cause nephrogenic system fibrosis and renal complications in patients with kidney disease [3]. Consequently, there is growing interest in imaging methods that can measure transvascular transfer in the brain without the use of contrast agents. For example with magnetization transfer-based MRI, where shifts between free and protein-bound water pools, measured from altered T_1_ relaxation time in the presence of off-resonance radiofrequency saturation (T_(1sat)_), may be indicative of leakage across the BBB [61]. MRI methods that enable quantification of transvascular water exchange based on perfusion and diffusion measurements have also been proposed [132]. These promising studies require further follow-up investigations to determine the sensitivity, specificity and clinical applicability of these contrast agent-free MRI methods for accurate and reliable detection of BBB permeability.

## Microscopic imaging of BBB dysfunction

As described above, MRI, CT and NIRF imaging can provide whole-brain information on BBB integrity, but spatial resolution is limited to macroscopic scales. However, microscopic assessment of BBB permeability is feasible with optical imaging in laboratory animals, in which small vessels are visualised on the cortical surface through a cranial window. With real-time fluorescence imaging, the extravasation of an impermeable fluorescent tracer from small cortical surface vessels has been measured in per- ilesional tissue as early as 30 min after cerebral ischaemia in rats [96]. An important advantage of intravital fluorescence imaging is the possibility of combined detection of cellular and molecular events that can provide insights into mechanisms that underlie BBB disruption. With longitudinal in vivo fluorescent angiography and parallel measurements of fluorescent markers of reactive oxygen/nitrogen species and cellular injury, Schoknecht et al. showed that propagation of vascular permeability within peri-ischaemic rat brain tissue is associated with free radical formation and progressive cell damage [109]. Even more detail can be obtained with two-photon microscopy, as recently demonstrated by Underly et al. who reported a central role of pericytes in early ischaemia-induced BBB disruption [127]. In transgenic mice with fluorescently labelled pericytes they observed that capillary BBB leakage after photo-thrombotic occlusion was preceded by MMP activation—visualised with a fluorescent probe for MMP-2/9 activity—occurring particularly at pericyte somata sites (Fig. 2a). Studies with transgenic mice where tight junctions are labelled with eGFP (Tg: eGFP-Claudin5 mice) have provided a detailed analysis of changes in structural components of the BBB in brain capillaries with in vivo two-photon microscopy in healthy and diseased brain (Fig. 4a) [62]. Dynamic imaging of tight junction turnover at the BBB in ischaemic stroke has demonstrated that although BBB function is impaired as early as 6 h after ischaemic stroke, TJs break down only 48 h after vessel occlusion in the transient middle cerebral artery occlusion (t-MCAO) model. These findings suggest that transcytosis impairs BBB function in the acute (within 6 h) phase of ischaemic stroke, whereas breakdown of TJs and increased paracellular permeability of CNS endothelium occurs in the late phase (24–48 h) following ischaemic stroke [62].

Taken together, various imaging methodologies have been established for qualitative and quantitative assessment of BBB permeability in stroke patients and animal models. Applications ranging from microscopic measurements in mice to whole-brain analyses in humans have improved our knowledge on patterns of BBB breakdown following cerebral ischaemia–reperfusion (Fig. 2). BBB permeability imaging methods are now being considered as complementary tools for diagnosis and outcome prediction in stroke patients, for example to guide reperfusion therapies that are accompanied by increased risk of haemorrhagic transformation. In vivo imaging methods that enable detection of additional disease markers at the level of the neurovascular unit can provide important complementary information on the pathophysiological consequences of cerebrovascular injury. For example, imaging strategies that allow in vivo detection of molecular targets, e.g. upregulated cell adhesion molecules with MRI [28], or multimodal SPECT/CT [125], have shown promise in assessment of (neuro) inflammation associated with stroke and BBB injury. Furthermore, prediction of ischaemic tissue fate has been shown to improve by including multiple imaging-based measures of ischaemia–reperfusion injury [15, 86]. The ongoing advances in imaging technologies will continue to enable original translational studies in experimental and clinical settings that will increase our understanding of the critical role of the BBB in stroke pathology and recovery, ultimately contributing to improved patient care.

## Cancer-mediated dysfunction of the blood-brain barrier

### Brain cancer biology

**Fig. 3:**
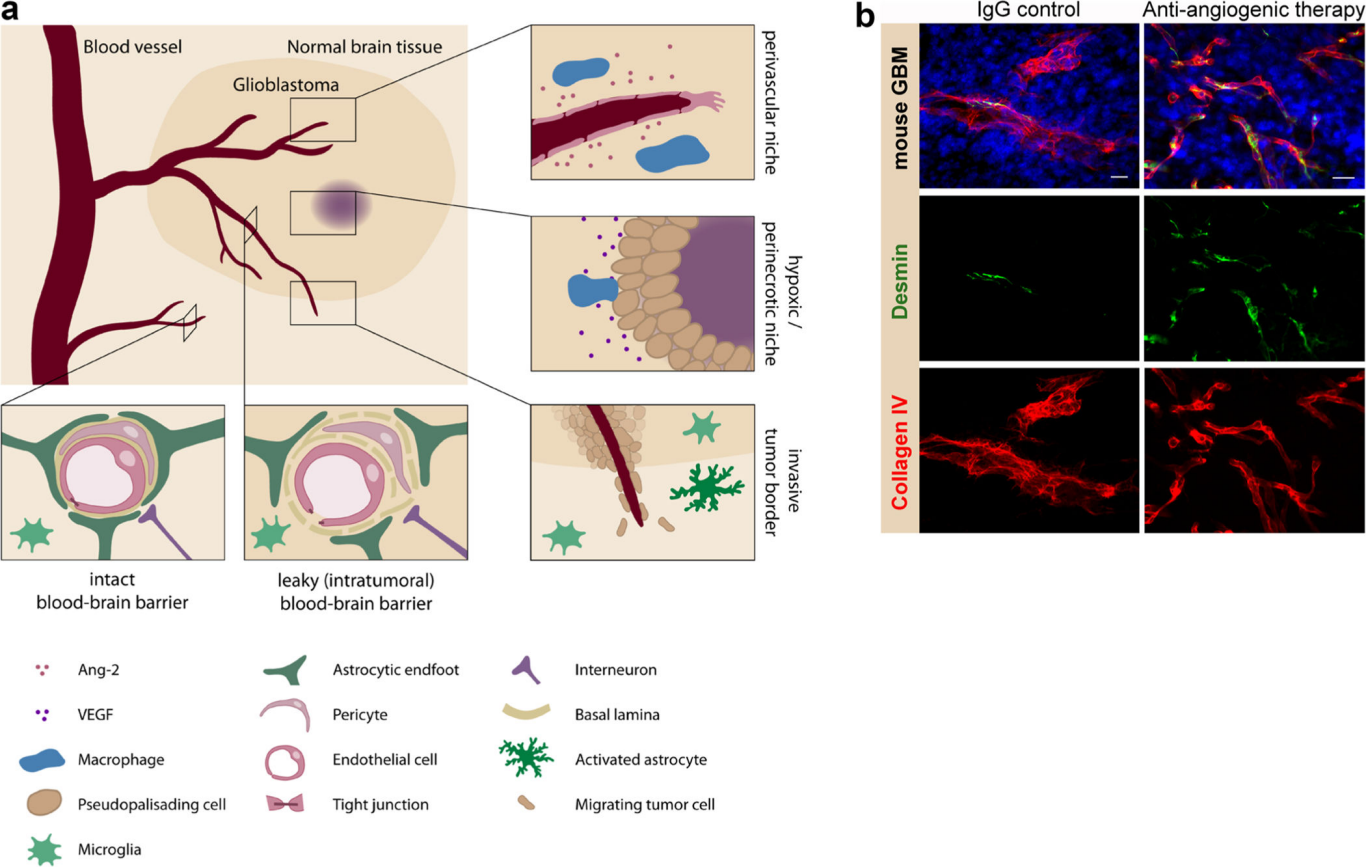
Schematic representation of the glioblastoma microenvironment. **a** Glioblastoma defining niches are displayed. *Perivascular niche:* Glioma and tumour stroma cells create a specialised vascular niche. Macrophages are the most abundant population that support the tumour growth by releasing proangiogenic factors. *Hypoxic/perinecrotic niche:* Necrotic areas are characterised by pseudopalisading cells and high levels of hypoxia which recruit tumour supporting macrophages. *Invasive tumour border:* Glioma cells are traversing the blood vessels. Peripheral blood vessels preserve intact blood–brain properties in contrast to the tumour vasculature which is leaky. **b** Combining anti-VEGF therapy with Ang-2 blockade acts synergistically on vascular normalisation. Immunofluorescence staining with antibodies directed against Collagen IV (red) and desmin (green) on vibratome sections of GL261 glioma bearing mice, untreated and after dual anti-VEGF (Aflibercept) and Ang-2 inhibition (Trebananib)

Many neurological disorders including brain tumours are almost always associated with increased vascular permeability, potentially leading to life threatening cerebral oedema [35]. Brain tumours represent a heterogeneous group of central nervous system (CNS) neoplasms that are classified into primary or secondary brain tumours according to their origin, i.e. intracerebral or spread from peripheral tumours [74]. The two most common types if intracranial neoplasms, brain metastasis and IDH wild type glioblastoma, account for > 70% of all brain tumours. Diffuse gliomas including IDH wild type glioblastoma, are the most common neuroectodermal tumours that arise in the white matter of the cerebral hemispheres and are characterised by infiltrative growth [74]. The progression of gliomas is highly angiogen- esis-dependent [98]. As a consequence of unphysiological angiogenesis the BBB is disturbed. VEGF signalling has been identified as the principal regulator of developmental and pathological angiogenesis which led to the development of anti-angiogenic cancer therapies in 2004 [39]. More recently, Angiopoietin (Ang)/Tie2 signalling which regulates vascular development, maturation and immediate vascular responses, has emerged as a novel vascular drug target in tumours, including glioma [106, 110]. Both angiogenic signalling pathways are well known to interfere with endothelial barrier properties [101, 106]. Disease progression of low-grade to high-grade glioma (WHO grade II–IV) is characterised by increased vascularization associated with BBB disturbances. BBB alterations are most prominent in glioblastoma multiforme (WHO IV, GBM), the most malignant brain tumour that is associated with high morbidity and poor median survival [74]. The current standard treatment for newly diagnosed GBM which includes maximal surgical resection followed by combined radiochemotherapy with temozolomide, does not extend overall survival beyond 15 months (for review see [98]). Anti-angiogenic therapy with Bevacizumab, a VEGF-targeting monoclonal antibody, is approved in a small number of countries for the treatment of recurrent GBM [98]. However, VEGF-targeting did not extend overall survival in first-line therapy [98]. Although intrinsic resistance mechanisms to anti-angiogenic therapy have been identified, unsuccessful drug delivery across the BBB may contribute to inefficient therapy [128]. The GBM microenvironment consists of highly specialised niches, such as the perivascular, the hypoxic-perinecrotic and the invasive niche at the tumour border which each display different BBB properties [45] (Fig. 3a). In the tumour centre, excessive vascular growth that is accompanied by severe hypoxia and necrosis due to elevated oxygen demands, leads to prominent BBB defects [98, 128]. At the tumour border, invasive glioma cells migrate along vessels that have yet a mostly intact BBB (Fig. 3a) [45, 137].

### Vessel architecture in glioma

GBM vessels are tortuous, disorganised and permeable with defective pericyte coverage and an abnormal basement membrane [98] indicative for a BBB breakdown. Characteristic features of the aberrant GBM microvasculature are hyperplasia and vascular sclerosis („glomeruloid tufts“with multilayered, proliferating ECs) [98]. VEGF signalling through its receptors (VEGFRs) was identified as the key driver for vascular growth in GBM more than 2 decades ago [39, 95]. VEGF is upregulated in hypoxic niches due to oxygen deprivation and secreted by pseudopalisading tumour cells which are aligned around necrotic cores [39, 95, 98]. In the hypoxic niche, vessels are obstructed and often collapsed as a result of vasogenic oedema. Vessels are hyperplastic which is associated with aberrant blood flow and a dysfunctional BBB. At the molecular level VEGF-induced BBB disruption in rodent GBM models is constituted by endothelialcell fenestrations, gaps and frequent numbers of caveolae [103]. Breakdown of the BBB is commonly characterised by a disturbed organisation of EC junctions rendering ECs permeable [137]. In peripheral vessels, VEGF-induced vascular permeability is mediated by VEGFR2 phosphorylation (Tyr 494 in rodents and Tyr 951 in humans, respectively) and VE-cadherin endocytosis (see [39]). In the brain, EC integrity is further dependent on the proper organization of tight junction molecules. A selective loss of claudins in GBM vessels (claudin-1, −3 in microvessels and claudin-5 in hyperplastic vessels) indicates their role as central mediators of barrier integrity and vascular permeability [137]. The chronic vascular hyperpermeability in GBM reflects the growth of structurally abnormal and immature vessels that are, among the described junctional defects, also deficient in pericytes [35] which will be further discussed in the section below. In addition, further cellular components of the neurovascular unit (NVU) that contribute to aberrant BBB features in GBM are astrocytes by means of loss of astroglial polarity [137]. Recently, invading glioma cells at the single cell level have been shown to be able to disrupt the BBB in mouse GBM via astrocyte-vascular uncoupling [133]. As mentioned above, Wnt/β-catenin signalling which plays an essential role in BBB formation and maintenance in the healthy CNS has been shown to regulate vascular quiescence and barrier function in GBM via upregulation of platelet-derived growth factor B (PDGF-B) and mural cell recruitment [100]. Wnt/β-catenin signalling hence represents a valuable therapeutic target for anti-angiogenic and oedema therapy.

## Angiogenic signalling and vascular integrity in gliomas

VEGF promotes neovessel formation in conjunction with EC-derived Ang-2 [39, 50, 101, 106]. Ang-2, an early GBM vessel marker [122], has recently-been identified as a resistance factor to VEGF monotherapy in GBM [110]. Upon Ang-2 upregulation, Ang-1-mediated Tie2 phosphorylation is prevented, resulting in immature, permeable vessels [101, 106, 110]. Improper NVU composition and functioning caused by high VEGF levels contribute to vessel permeability in GBM, and upon recurrence often lead to life threatening oedema [98]. Studies following VEGF monotherapy or combined VEGF/Ang-2 targeting in preclinical GBM models demonstrated a more normalised vasculature [106, 110]. Previously immature glioma vessels with a defective BBB/NVU that lack pericytes are consequently reverted to a more “normal“, pericyte-rich phenotype as a consequence of increased Tie2 signalling (Fig. 3b; [110]). Activation of the Ang-1/Tie2 axis is critical in order to achieve a mature vasculature during, both developmental and pathological angio- genesis [101, 106]. Pericytes have previously been shown to be essential for proper BBB functioning [27]. Evidence in an Ang-2 gain-of-function (GOF) mouse model demonstrated a leaky BBB as the result of Ang-2-mediated pericyte-deficiency, defective interendothelial junctions, increased numbers of vesicles and a disrupted glycocalyx [43]. Similarly, in the peripheral tumour vasculature, Ang-2 overexpression decreased endothelial integrity, while Ang-2 blockade improved EC junctions and basement membrane contacts of metastasis-associated lung capillaries [51]. In a preclinical GBM model (GL261) in Ang-2 GOF mice, increased IgG permeability and hypoxia were evident and reversed upon anti-angiogenic VEGF and Ang-2 therapy [110] or by using a bi-specific Ang-2/VEGF-targeting approach [106]. Moreover, in other models with a disrupted BBB such as cerebral ischaemia, activation of Tie2 via inhibition of the vascular-endothelial phoshotyrosine phosphatase (VE-PTP) which interferes with Tie2 phosphorylation in the absence of VE-cadherin, promoted junctional stabilisation of cerebral vessels [43]. VE-PTP inhibition has been shown to be effective in ocular diseases, metastasis and permeability models and clinical trials are currently pursued for macula oedema [18]. In GBM, VE-PTP inhibition may represent an alternative strategy for oedema control by steroids (e.g. dexamethasone) as corticosteroid therapy is associated with undesired off-target effects in the immune system [94] that may be counterintuitive in cancer immunotherapy.

## Role of the BBB in glioma-associated inflammation/immune cell recruitment

The brain vasculature (BBB) not only controls the passage of metabolites but also controls leukocyte trafficking. Although the healthy brain restricts the trafficking of immune cells, in GBM immune cells are important constituents of the tumour microenvironment as they penetrate the dysfunctional GBM vasculature. The tumour pro-angiogenic vasculature thereby prevents the recruitment of tumour-reactive T lymphocytes and fosters an immunosuppressive microenvironment that allows gliomas to evade host immune-surveillance [84]. Sustained angiogenesis and immunosuppression are hallmarks of cancer [46] and the abundance of tumour-associated macrophages (TAMs) has been associated with poor clinical outcome [98]. Macrophages secrete a plethora of cytokines and growth factors (among them VEGF) that contribute to tumour progression and vascular permeability [98]. Myeloid-derived VEGF and signalling through VEGFR1 thereby contribute to GBM progression [58, 89]. Recently, Ang-2/Tie2 signalling has been linked to cancer inflammation as well as it promotes the recruitment of pro-angiogenic, M2-polarised macrophages that contribute to therapy resistance [101, 106, 110]. Furthermore, a subpopulation of macrophages in the perivascular tumour niche characterised by Tie2 receptor expression known to contribute to tumour angiogenesis have recently been associated with the metastatic cascade [68]. In mammary tumours perivascular macrophages promote the transient opening of tumour blood vessels by means or VEGF secretion to facilitate cancer cell dissemination (see [68]). These findings may in part explain the hyperpermeable nature of tumour vasculature that is described as spatially and temporally heterogeneous. Such findings may also translate to glioma where aforementioned disturbances of the BBB are prominent in some regions (GBM core) while the NVU is functional in other regions (invasive zone) (Fig. 3a). Co-targeting of vascular integrity and pro-angiogenic, innate immune cells by angiogenic inhibitors (VEGF, Ang-2) already showed improved therapeutic efficacy in GBM models [90, 106, 110]. A normalised vasculature is also permissive for the recruitment of cytotoxic T lymphocytes thereby creating a tumour microenvironment that promotes anti-tumour targeting by cytotoxic T lymphocytes that secrete inflammatory cytokines upon combined anti-angiogenic and checkpoint therapy as recently shown in different tumour models including GBM [4, 108]. Similarly, Ang-2 and Tie2 co-targeting has been shown to be superior to either targeting Ang-2 (inhibition) and Tie2 (activation) alone as recently demonstrated by Park and colleagues [90]. A novel Ang-2-binding and Tie2-activating antibody (ABTAA) induced tumour vessels normalisation, reduced brain oedema, and changed innate immune cells towards an M1 phenotype in an orthotopic glioma model [90]. The glioma vessel normalisation further enhanced intra-toumoral Temozolomide delivery [90]. Combined Tie2 activation and Ang-2 inhibition thus offers a novel therapeutic approach to elicit a favourable tumour microenvironment and enhance the delivery of chemothera-peutics into tumours. The restoration of Tie2 signalling may also have implications for other disorders associated with defective BBB, i.e. stroke [43], epilepsy [11] and metastasis whereby disease onset may be prevented by vascular stabilising agents.

## Preventing brain metastasis by stabilising the blood–brain barrier

A rising incidence of brain metastases that commonly arise from cancers of the lung, breast and skin increase the demand for novel treatment to cure patients [98]. Tumour cells that have entered the bloodstream need to penetrate the BBB in order to get access to the brain parenchyma. The partial disruption of the BBB leads to increased trespassing of molecules and cells and thereby potentially facilitating the colonisation of tumour cells into the brain. Treatment of mice with the neutralising Ang-2 peptibody Trebananib for instance has been shown to prevent changes in the BBB integrity and to inhibit breast cancer cell colonisation to the brain [8]. However, a heterogeneous blood–tumour barrier or BBB permeability determines the drug efficacy in experimental brain metastases of breast cancer. Data from Lock-man et al. indicate that the BBB remains partially intact in experimental brain metastases and thus impair drug delivery which demonstrates a need for brain permeable molecular therapeutics [73]. In this context, the impact of the BBB integrity for targeted therapy in different stages of brain metastases has recently been investigated [88]. The study suggests, that only drugs designed to fully penetrate the BBB are therapeutically efficacious [88].

On the other hand, interference with VEGFR1 signalling inhibited monocytic cell migration into orthotopic brain tumours and subsequently slowed tumour growth [58, 89]. Of interest, a recent meta-analysis of clinical trials provided evidence that VEGF-inhibition is associated with fewer brain metastases in patients diagnosed with adenocarcinomas of the lung [54]. Therefore, it is of considerable interest to investigate whether the local destabilisation of the BBB acts as a prerequisite for successful formation of brain metastases by supporting the development of pre-metastatic perivascular niches and if so, whether the stabilisation of the BBB by pharmacological inhibitors is sufficient to prevent the formation of brain metastases.

## Blood–Brain Barrier Dysfunction and Immune Cell Trafficking in neuroinflammation

Multiple sclerosis (MS) is the most common cause of nontraumatic neurological disability affecting approximately 2.5 million people worldwide. Although the pathogenesis leading to MS is not well understood, multiple studies have demonstrated the autoimmune nature of the disease in which self-reactive T cells specific for myelin proteins initiate an inflammatory cascade resulting in neuro-inflammation, demyelination and axonal damage [67]. The extensive heterogeneity in both disease course and pathological features seen in MS patients suggests that multiple pathways contribute to disease pathogenesis. The majority of MS patients exhibit a relapsing–remitting disease course (RRMS) characterised by the occurrence of focal inflammatory lesions within the CNS with a leaky BBB detectable by MRI and focal regions of demyelination [27].

The pathogenic mechanisms of MS have been extensively studied using the animal model experimental autoimmune encephalomyelitis (EAE), which is induced either by the activation or adoptive transfer of CD4^+^ myelin-specific T cells. Among various immune cell subtypes, CD4^+^ Th17 (IL-17-producing) and Th1 (IFN-γ-producing) lymphocytes are the most prominent pathological immune cell types in both MS/EAE. These cells are distinguished by secretion of unique effector cytokines that have distinct effects in either promoting axonal damage and oligodendrocyte death or inducing BBB dysfunction [123]. The conventional Th17 cells generated in the presence of TGF-β1 and IL-6 are non-pathogenic during EAE, since they are involved in the maintenance of mucosal surface homeostasis and antibacterial defence. However, several inflammatory cytokines such as IL-1β, IL-23, and TGF-β3 promote generation and maintenance of highly pathogenic Th17 cells during EAE. These pathogenic Th17 cells express both RORyt and T-bet transcription factors, produce both IL-17A and IFN-γ and are preferentially recruited into the CNS (reviewed in detail in [115]). Other subsets of CD4^+^ T cells, such as Th1 and Th9 also contribute to the development of neuro-inflammation and autoimmunity. The adoptive transfer of MOG-specific CD4^+^ Th1 and Th9 cells induces EAE in C57BL/6 recipients [34, 115]. Moreover, IL-9 is required for mast cell activation, which has been shown to degrade myelin during CNS inflammation [34]. While both Th1 and Th17 effector CD4^+^ T cell subsets are capable of inducing EAE in adoptive transfer studies, Th1 and Th17 cells show unique temporal profiles when they infiltrate the CNS during EAE. Th17 cells show the highest concentration in the spinal cord at 7 days post-immunisation and decrease to baseline by day 10 in EAE. In contrast, Th1 lymphocytes are low at day 10 and escalate in number in the EAE spinal cord by day 14 [85]. Consistent with these observations, adoptive transfer of Th17 cells that are differentiated ex vivo induces more rapid clinical presentation of disease, as compared to adoptive transfer of Th1 cells differentiated ex vivo [105]. The potential explanation for these unique temporal features of CNS entry will be discussed below.

While the main emphasis has been placed on myelin-specific CD4^+^ T cells in MS/EAE pathogenesis, recent studies suggest that neutrophils are also critical for disease. In EAE, neutrophils comprise a significant percentage of CNS-infiltrating cells prior to disease onset and relapse [91]. When neutrophils are depleted prior to, but not after, there is an improvement in disease onset or relapse, suggesting that neutrophil function is important during the initial formation of lesions [111]. Neutrophils in the blood of RRMS patients exhibit a primed phenotype, and both their number and activity increase during relapses. Similarly, MS patients have a higher neutrophil-to-lymphocyte ratio in the blood compared to healthy controls, and the ratio increases with the occurrence of relapse and worsening disability [111]. However, neutrophils are not a pronounced feature of CNS pathology in MS patients. One potential explanation is that neutrophils have a short half-life and may only contribute to disease at specific stages, i.e. prior to onset or relapses. Nevertheless, they play an important role in the initial BBB dysfunction [7] (see below).

## Mechanisms of immune cell interactions with the CNS vasculature in neuro-inflammation

In MS and EAE, leukocytes or other immune cells migrate across multiple pathways to reach the CNS parenchyma, including the BBB and choroid plexus. The transmigration across the BBB is a very dynamic process and depends on several sequential, yet interdependent steps constituting (1) tethering, (2) rolling, (3) crawling, (4) arrest, (5) diapedesis of immune cells across the ECs and (6) destruction of the basal lamina [20]. Leukocytes may also infiltrate the CNS via the blood–cerebrospinal fluid route through an epithelial barrier present within the choroid plexus. Intravital microscopy analysis of encephalitogenic T cell interactions with inflamed brain and spinal cord microvessels has shown that interactions of P-selectin glycoprotein ligand (PSGL-1) with P/E-selectin mediates the initial rolling and tethering of T cells [59]. However, deficiencies in E- and P-selectin or PSGL-1 do not protect mice from EAE, suggesting redundant roles for these proteins in neuro-inflammation [31]. Inflamed CNS endothelial cells upregulate expression of both the intercellular adhesion molecule 1 (ICAM-1) and vascular cell adhesion molecule 1 (VCAM-1). The respective ligands, α_L_β_2_ [lymphocyte function-associated antigen 1 (LFA-1)], and α_4_β_1_ [very late antigen 4 (VLA-4)] integrins, are expressed on encephalitogenic CD4^+^ T cells. Multiple studies have shown that LFA-1-ICAM-1 and VLA-4-VCAM-1 interactions are critically involved in the firm arrest of CD4^+^ T cells onto the inflamed CNS vessels or primary brain EC monolayers [20]. Moreover, LFA-1-ICAM-1 interactions dictate the polarisation and crawling of CD4^+^ T cells onto the inflamed CNS vessels. Interestingly, α_4_β_1_integrin-VCAM-1 interaction preferentially arrests encephalitogenic Th1 cells onto the spinal cord blood vessels suggesting that Th1 cells preferentially use α4-integrin for transmigration across the BBB [105]. The anti-α4-integrin antibody, natalizumab has been approved for the treatment of relapsing–remitting MS suggesting that the arrest step is critical for disease pathogenesis. However, live cell imaging studies of transendothelial migration across ICAM-1 and ICAM-2-deficient brain endothelial monolayers have shown that CD4^+^ T cell may undergo diapedesis in an ICAM-independent manner [1], suggesting that other adhesion molecules may function in a redundant manner.

Additional cell adhesion molecules such as activated leukocyte cell adhesion molecule (ALCAM) and melanoma cell adhesion molecule (MCAM) also control transmigration of the CD4^+^ and CD8^+^ T cells across the BBB and CNS autoimmunity [22, 65]. Th17 autoreactive T cells express very high levels of MCAM and antibody-mediated blockade of MCAM reduces CNS autoimmunity [65]. Antibody-mediated inhibition of ALCAM reduced diapedesis of human CD4^+^ Th1 but not of Th17 cells across a human BBB *in vitro.* However, ALCAM play a significant role in rolling, adhesion, and diapedesis of human monocytes across the human BBB suggesting that distinct cell adhesion molecules may contribute to selective interactions of distinct classes of immune cells with the inflamed CNS endothelium in neuroinflammation [69]. ALCAM deficient mice developed a more severe EAE due to a reduced expression of BBB junctional proteins. ALCAM is associated with the assembly of tight junction molecules that explains the increased permeability of CNS blood vessels in mutant animals [69]. Therefore, ALCAM may also function to maintain BBB integrity.

Once arrested, CD4^+^ T cells start crawling on inflamed CNS microvessels to search for sites of diapedesis. Immune cells can cross the BBB through migration via the paracellular route due to breakdown of EC tight junctions (TJs) or the transcellular route, mediated by an increase in the number of caveolar vesicles that transport cells and large molecules including antibodies across CNS blood vessels [42]. How is Th17 versus Th1 CD4^+^ T cell trafficking across a dysfunctional neurovascular barrier coordinated with degradation of tight junctions and increased caveolar transcytosis during MS/EAE? Previous studies have shown that Th17 cells show the highest concentration in the spinal cord at 7 days postimmunisation that decreases to baseline by day 10 in EAE. In contrast, Th1 lymphocytes are low at day 10 and escalate in number in the EAE spinal cord at day 14 [85]. We have recently found that caveolin-1 (Cav-1), a protein essential for the formation of caveolae, is not required for either tight junction degradation at the BBB or early development of clinical EAE; however, it is required for transmigration of a subset of Th1 cells across the BBB [76]. Why do Th1 lymphocytes preferentially cross the BBB via caveolae in EAE? Cell adhesion molecules such as ICAM-½ and VCAM-1 are enriched in caveolae and support transcellular migration of immune cells by engaging infiltrating cell podocytes [1, 20]. Upon attachment of the CD4^+^ T cells to the apical surface of inflamed brain ECs, ICAM-1 and VCAM-1 are clustered around transmigrating CD4^+^ T cells, resulting in the formation of caveolae enriched with actin filaments [1, 20]. Increased levels of ICAM-1 on the apical surface of primary mouse brain microvascular cell monolayers may promote transcellular migration of CD4^+^ T cells possibly because of high occupancy of its receptor LFA-1 on CD4^+^ T cells [1, 20]. Th17 cells rely primarily on an αLβ2/ICAM-1 interaction [105, 123], whereas Th1 cells employ an α4/ VCAM-1 complex for their migration [41]. Thus, selective interactions between these adhesion molecules may direct Th1 cells more effectively toward caveolae as compared to Th17 when crossing the CNS vessels in neuro-inflammation.

**Fig. 4:**
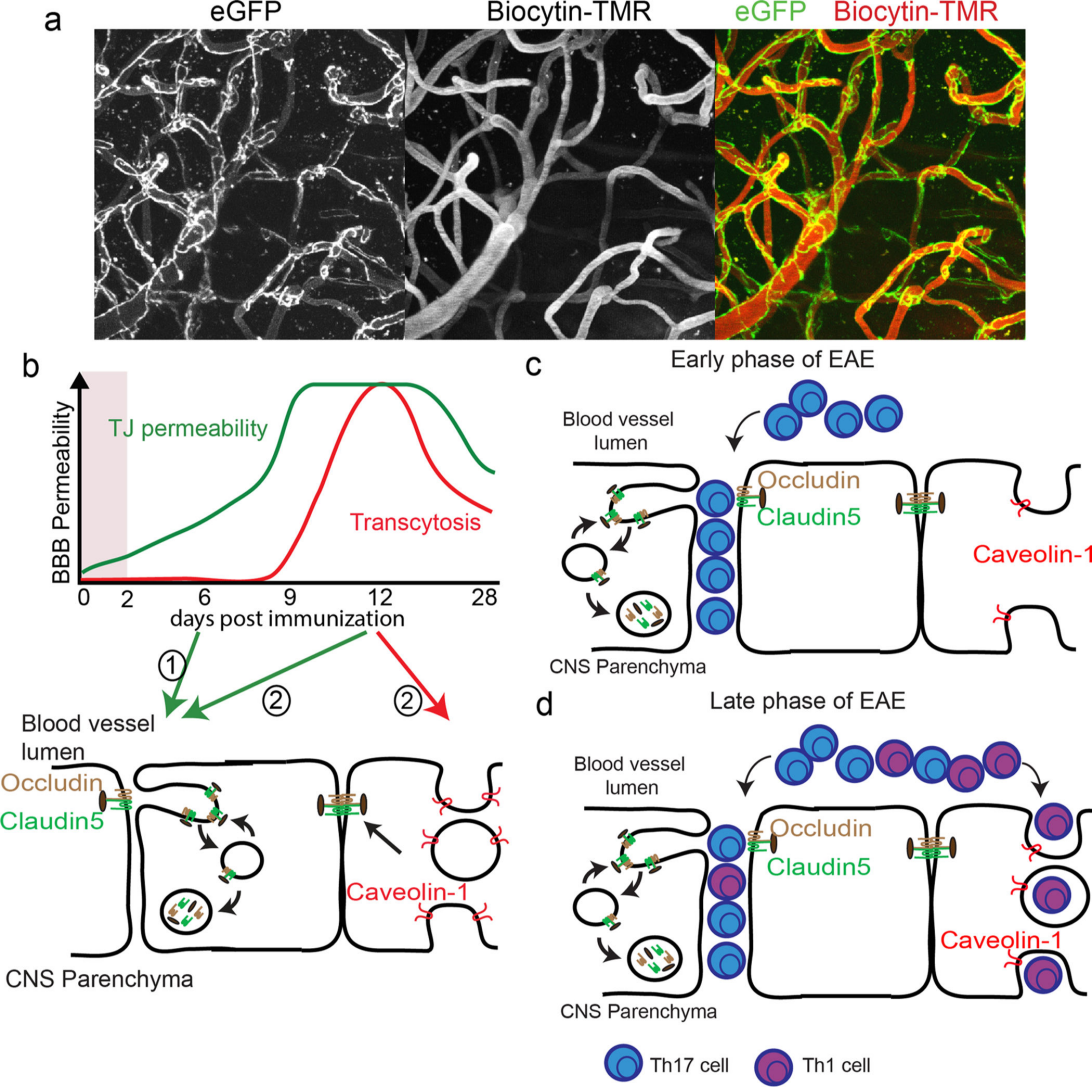
Schematic presentation of BBB breakdown and lymphocyte trafficking across a damaged BBB in neuroinflammation. **a** Transgenic mice that allow visualisation of endothelial tight junctions (*Tg eGFP::Claudin*-*5*) with intravital two-photon microscopy. The image shows cortical blood vessels that have eGFP in tight junctions (green). Biocytin-TMR tracer (red) was injected into the tail vein of mice to visualise the vascular tree. This tracer also allows measurement of BBB permeability. **b** A working model of BBB disruption during EAE. Increased paracellular BBB permeability (green line) occurs prior to onset and persists throughout EAE. Transcellular BBB permeability (red line) only transiently elevates at acute disease. Para-cellular permeability is due to rapid remodelling of TJ proteins, while transcellular permeability results from enhanced caveolar trafficking. **c** During the early phase of MS/EAE progression, there is an initial breakdown of endothelial cells TJs at the BBB that causes an increase in paracellular permeability. This allows the preferential entry of Th17 lymphocytes (blue) in the early phase of the disease, although some Th1 lymphocytes (purple) also enter through this route. **b** The increase in caveolar transport (transcellular permeability) through upregulation of Caveolin-1 is observed only during the late phase of neuroinflammation at the peak of the disease. Th1 lymphocytes (purple) preferentially use caveolae to cross the BBB through enhance transcellular permeability. TJ remodelling in both phases of the disease involves formation of membrane invaginations (protrusions) that fuse with EEA1^+^ endosomes. However, TJ remodelling is independent of caveolae suggesting that in MS/EAE, similar to BBB development, there are distinct mechanisms that impair paracellular versus transcellular barrier properties of the CNS vasculature

Other cell adhesion and junction proteins such as PECAM-1, CD99, claudin-5, VE-cadherin, VE-PTP and JAMs have been shown to play an important role in regulation of paracellular migration across the BBB. CD99 is a crucial regulator of diapedesis of leukocytes through the blood vessel wall. Genetic inactivation of CD99 causes neutrophil accumulation between endothelial cells and the basement membrane in inflamed peripheral post capillary venules and impaired leukocyte attachment to the luminal surface of the vessel [42]. CD99 promotes leukocyte attachment to the endothelium in inflamed vessels by a hetero- philic ligand. In addition, CD99 binds to the paired immunoglobulin-like receptors (PILRs) on neutrophils to increase the shear resistance of the neutrophil attachment to ICAM-1 [42]. CD99 can also interact with PECAM-1 to promote immune cell migration across the blood vessels. Blockade of CD99 or CD99L2 function, or genetic inactivation of PECAM-1 traps neutrophils between endothelial cells and the underlying basement membrane in vivo in the inflamed peripheral blood vessels. CD99 is also critical for lymphocyte transmigration across a human BBB model in vitro. Blocking CD99 in vivo ameliorates EAE and decreases the accumulation of CNS inflammatory infiltrates, including dendritic cells, B and T cells. Anti-CD99 therapy is effective to block relapse when administered therapeutically after disease symptoms had recurred [135]. These findings indicate an important role for CD99 in the pathogenesis of CNS autoimmunity and suggest that it may serve as a novel therapeutic target for controlling neuro-inflammation. CD99, PECAM-1 and VE-cadherin are enriched in an endothelial-specific subcellular compartment called the lateral border recycling compartment. The lateral border recycling compartment has been proposed to function as the subcellular organelle where rapid remodelling of cell junctional proteins happens in brain ECs as they interact with transmigrating monocytes in vitro [136]. These organelles may be homologous to dynamic TJ protrusions that several groups have observed by two photon imaging in either epithelial cells of the gut [78] or endothelial cells in the brain [62] and spinal cord (Fig. 4) under inflammatory conditions. This compartment has been shown to support both paracellular and transcellular migration in vitro; however, it is unclear if it this compartment has any role in transmigration across the BBB in vivo.

During inflammation, activated T cells can cross the endothelial basement membrane, but additional signals are required to break down the glia limitans to allow leukocytes to infiltrate the CNS parenchyma. After crossing the endothelial vessels of the BBB, CD4^+^ T cells encounter the glial (glia limitans) basement membrane, and breaching this acellular structure represents a challenge for CD4^+^ T cells to arrive at the CNS. The endothelial basement membrane at the BBB is characterised by the presence of both laminin α_4_ and α_5_. Encephalitogenic CD4^+^ T cells cross the endothelial basement membrane through α_6_β_1_-integrin-laminin α_4_ interactions, whereas laminin α_5_ inhibits their migration [115]. In contrast to the endothelial basement membrane, the glia limitans is enriched with laminin α1 and α2. Since encephalitogenic CD4^+^ T cells do not interact with laminin α1 and α2, they depend on secretion of matrix metalloproteinases (MMPs) to degrade this basement membrane in order to enter the CNS. It is hypothesised that matrix metalloproteinases (MMPs) secreted by neutrophils contribute to this step in the pathogenesis of MS. Various types of MMPs, such as MMP-2, −7, −8, and −9, have been identified in the CSF and MS/EAE lesions. MMP2 and MMP9 expression are specifically increased during EAE, and their activity is positively correlated with the migration of CD4^+^ T cells across the glia limitans (see [91] for more details). One of the MMP2/9 targets is β-dystroglycan, a receptor which anchors astrocytic endfeet to the parenchymal basement membrane, leading to secretion of chemokines by the astrocytes at the glia limitans. Therefore, neutrophils may specifically alter the glia limitans to promote the final step of CD4^+^ T cell entry into the CNS in neuro-inflammation [91].

## How do immune cells affect the blood–brain barrier in neuro-inflammation

Several TJ proteins such as claudin-3, −5, −12 and occludin, that normally restrict paracellular movement of molecules across the BBB, are disrupted in neuro-inflammatory diseases including MS/EAE. Endothelial TJ degradation precedes overt lesion formation in MS, correlates with clinical EAE severity and promotes leukocyte migration via the paracellular route [6, 38]. On the contrary, overexpression of the TJ-associated protein claudin-1 is protective for EAE (see [35]). TJs are highly dynamic in vivo and their rate of turnover is increased after inflammation in both gut epithelial cells and brain EC following ischaemic stroke or EAE [62, 76, 78]. Although BBB disruption promotes neuroinflammation by enabling inflammatory proteins, antibodies and leukocytes to access the CNS, it has remained elusive how dynamic remodelling of EC TJs regulates immune cell trafficking into the CNS during EAE progression. We have recently shown using intravital two-photon microscopy in *Tg eGFP-Claudin5*^*+/–*^ mice, in which EC TJs are labelled with enhanced green fluorescent protein (eGFP) to visualise dynamic changes in TJs during EAE that dynamic remodelling of TJs and paracellular BBB leakage increase prior to the onset of EAE and remain high throughout disease (Fig. 4b) [76].

In addition to the presence of TJs, BBB permeability is also controlled via non-clathrin-coated caveolae that are highly enriched in Cav-1. The density of endocytotic vesicles within CNS endothelium and the expression of Cav-1 increase during acute MS/EAE [76], suggesting that endothelial caveolae may provide a migratory route for myelin-specific T cells into the CNS (Fig. 4b). Caveolae cluster adhesion molecules are essential for interactions between immune cells and endothelium [20]; moreover, they also regulate turnover of TJ proteins in both epithelial and ECs in vitro induced by inflammatory cytokines [78, 119]. These studies suggest that caveolae may play an essential role in EAE pathology by orchestrating both TJ degradation and paracellular migration of immune cells across the BBB as well as promoting adhesion and migration of immune cells via the transcellular route. In support of this hypothesis, mice lacking Cav-1, a protein essential for caveolae formation, are protected from EAE and develop minimal clinical manifestations with very few CD4^+^ T cells infiltrations into the CNS [138]. However, dynamic remodelling of TJs at the neuro-vasculature is not changed in Cav-1^–/–^ mice during EAE progression, suggesting that caveolae do not play a role in remodelling of EC junctions in EAE. Moreover, Cav- 1^–/–^ mice have a selective decrease in the number of Th1, but not Th17 lymphocytes in the CNS. Therefore, caveolae-independent remodelling of TJs facilitates Th17 lymphocyte transmigration across the BBB in neuro-inflammation, whereas caveolae regulate the entry of Th1 lymphocytes into the CNS (Fig. 4c) [76].

What controls TJ remodelling and degradation during inflammation in vivo? Caveolae have been proposed to promote TJ endocytosis in both CNS and non-CNS ECs in vitro, as well as in non-CNS ECs in vivo [78, 119]. We have found that dynamic TJ protrusions are highly prevalent prior to EAE onset consistent with the proposed role for neutrophils in promoting early BBB breakdown by secretion of proteolytic enzymes (Fig. 4b) [41, 91]. In EAE, an increase in BBB permeability is associated with the early influx of neutrophils into the CNS. Depletion of neutrophils helps preserve BBB integrity, suggesting that neutrophils play a role in early BBB breakdown [91]. Neutrophils have also been correlated with BBB leakage in an acute MS lesion and their presence correlates with observations of dynamic TJ protrusions at the BBB prior to disease onset. Although the exact mechanisms of neutrophil-mediated BBB breakdown are still unknown, it is hypothesised that matrix metalloproteinases (MMPs) secreted by neutrophils contribute to this step of disease pathogenesis [91].

Another potential mechanism that may control the rate of TJ degradation in CNS ECs could be an enhanced endo-cytotic or macro-pinocytotic remodelling/degradation of junctional proteins in response to inflammatory cytokines secreted by immune cells or microglia that are present in EAE. IL-17 and IL-22 cytokines produced by Th17 cells disrupt BBB tight junctions in vitro and in vivo [57]. IL-17A induces NADPH oxidase- or xanthine oxidase-dependent reactive oxygen species (ROS) production that in turn activates myosin light chain kinase (MLCK) leading to down-regulation of occludin [52]. Blocking either ROS formation, myosin light chain phosphorylation, or applying IL-17A-neutralising antibodies prevents IL-17A-induced BBB disruption [52]. Treatment of EAE mice with ML-7, an MLCK inhibitor, results in reduced BBB disruption at the spinal cord and lymphocyte infiltration and is associated with reduced clinical characteristics of EAE [52].

Encephalitogenic CD4^+^ Th17 cells can also indirectly breakdown the BBB. Th17 cells induce expression of CXCL1 and CXCL2 in the spinal cords which are two chemokines that promote BBB breakdown via mobilisation of polymorphonuclear leukocytes (PMN) [19].

Th1 CD4^+^ T cells also have a negative impact on BBB function. cells IFN-γ, a cytokine predominantly produced by Th1 T cells, increases expression of ICAM-1, VCAM-1, MAdCAM-1, H2-K^b^ and I-A^b^ molecules on brain endothelial cells and induces transendothelial migration of CD4^+^ T cells from the apical (luminal) to the basal (abluminal) side of the endothelial monolayer. IFN-γ favours the transcellular route of CD4^+^ T cells migration by promoting caveolar transport [116]. In culture, IFN-γ induces internalisation and re-localization of VE-cadherin, PECAM-1, ZO-1 and Claudin-5 in the endothelial cells, which affects the migration of CD4^+^ T cells. IFN-γ produced during inflammation could contribute towards disrupting the BBB and promoting transendothelial migration of CD4^+^ T cells [116]. The inflammatory cytokines IL-1β and TNF-α and CCL2 are elevated in the acute phase of MS and EAE and can enhance paracellular barrier permeability [14]. CCL2 has been shown to promote degradation of claudin-5 in a Cav-1-dependent manner in brain ECs in vitro [119].

Finally, matrix metalloproteinases (MMPs) secreted by neutrophils also contribute to BBB damage during MS/EAE pathogenesis. The activity and expression of MMP-9 is increased in serum, CSF, and active lesions in MS and has been associated with BBB breakdown in mice. MMP-9 can damage several BBB proteins such as adherens and tight junctions. Neutrophils can also produce ROS, which is known to disrupt junctional proteins of the BBB endothelium, leading to increased permeability.

## Blood–brain barrier pathology in Alzheimer’s disease

Alzheimer’s disease (AD) is a chronic neurodegenerative disorder characterised by neuronal degeneration, gliosis, and amyloid beta (Aβ) accumulation, leading to cerebral amyloid angiopathy (CAA), senile plaque formation and the development of neurofibrillary tangles containing hyperphosphorylated neuronal tau protein [99]. AD pathology is also characterised by chronic brain inflammation, with microglial cells implicated in the accumulation of Aβ and neuronal damage [47].

BBB dysfunction also contributes to the onset and progression of AD [83, 143]. For example, BBB transport systems are significantly altered in AD patients compared to controls [83]. The glucose transporter GLUT1 is expressed at a lower level in the brain capillaries of AD patients and mouse models of AD, and GLUT1 deficiency leads in ECs leads to the loss of TJ proteins and BBB dysfunction in mouse models of AD [81, 83]. Glucose uptake at the BBB is disrupted in patients with mild cognitive impairment (MCI) and this may precede neurodegeneration and conversion to AD [83]. The expression of Aβ peptide transporters in ECs also changes during AD [149]. The expression of low-density lipoprotein receptor related protein 1 (LRP1), which mediates the efflux of Aβ from the brain to the periphery, is significantly reduced in the brain ECs of AD patients, whereas the expression of endothelial RAGE increases, promoting Aβ influx back into the CNS and favouring Aβ accumulation in the brain [83]. Aβ oligomers can directly induce the expression of RAGE in ECs, further contributing to the altered expression and function of Aβ transporters [83].

Aβ is a major contributor to BBB dysfunction in AD and Aβ deposits in the vasculature enhance BBB permeability in the AD brain. Previous studies have shown that CAA promotes the degeneration of smooth muscle cells, pericytes and ECs, leading to BBB breakdown [36]. Aβ disrupts TJs and increases vascular permeability by inhibiting the expression of TJ proteins and inducing the expression of matrix metalloproteases (MMPs), which may in turn degrade TJ components [63, 131]. Furthermore, the expression of TJ proteins is significantly lower in patients with capillary CAA, leading to increased vascular permeability in the AD brain [21]. Experimental evidence suggests that tau pathology also disrupts the integrity of the BBB, thus both tau and Aβ may induce BBB dysfunction, promoting neurodegeneration and cognitive impairment [13].

Changes in the extracellular matrix (ECM) that forms the BBB basement membranes are also observed in AD patients, suggesting they may contribute to BBB dysfunction in AD [143]. MMPs such as MMP-3 and MMP-9 are more abundant in the cerebrospinal fluid (CSF) of AD patients than controls, and may be responsible for ECM degradation at the BBB level in AD [121]. Secreted MMPs can digest the endothelial basal lamina and TJ scaffold proteins, which are necessary for BBB integrity, and may lead to BBB injury in AD. Furthermore, Aβ1–42 oligomers induce the expression of MMP-2 and MMP-9 in brain ECs, and MMPs released by ECs may contribute to basement membrane degradation and BBB dysfunction [131]. Pathological changes in the structure and organization of basement membrane ECM proteins may favour the migration of circulating leukocytes, which represent a key inflammatory process in AD [143, 144].

Cellular components of the neurovascular unit (NVU), such as pericytes and glial cells, may also contribute to BBB permeability in AD. Recent studies have shown that AD patients lose significant numbers of pericytes in the cortex and hippocampus compared to controls, suggesting that pericyte dysfunction contributes to BBB disintegration in AD (for review see [83]). Pericyte deficiency leads to BBB damage, cognitive impairment, increased Aβ deposition and tau pathology, further suggesting a predominant role of pericyte dysfunction in AD pathogenesis (for review see [83]). The expression of *APOE4* (a major genetic risk factor for AD), but not *APOE3,* leads to pericyte loss in AD, which correlates with the magnitude of BBB degradation to plasma proteins [44]. Recent studies have shown higher CSF levels of soluble platelet-derived growth factor receptor β (sPDGFRβ), a marker of pericyte injury, correlating with increased BBB permeability in the hippocampus of MCI patients compared to controls, indicating that pericyte damage is a key mechanism leading to cognitive impairment (for review see [83]).

Most of the glial cells surrounding the parenchymal basal membrane of brain microvessels are astrocytes, and their dysfunction may also contribute to BBB breakdown in AD. Changes in astrocyte morphology have been observed near blood vessels with Aβ deposits in the AD brain, whereas experimental studies have shown the retraction and swelling of astrocyte endfeet surrounding vascular Aβ deposits and the downregulation of GLUT1 and lactate transporter expression [81]. Aβ pathology disrupts the perivascular sheath of astrocyte processes by reallocating AQP4, and this may induce astrocyte depolarization [141]. During inflammatory responses, astrocytes may secrete several types of cytokines and chemokines, such as interleukin (IL)-1, CXCL1, IL-6, IL-8 (CXCL8), monocyte chemoattractant protein 1 (MCP-1/CCL2), interferon gamma-induced protein 10 (IP-10/CXCL10) and macrophage inflammatory protein 1 alpha (MIP-1β/CCL3), which may attract circulating leukocytes inside the brain and fuel a chronic inflammatory process. Activated perivascular microglial cells may also secrete a plethora of inflammatory mediators including cytokines and chemokines, and may actively promote neuroinflammation and leukocyte recruitment into the AD brain [143].

**Fig. 5:**
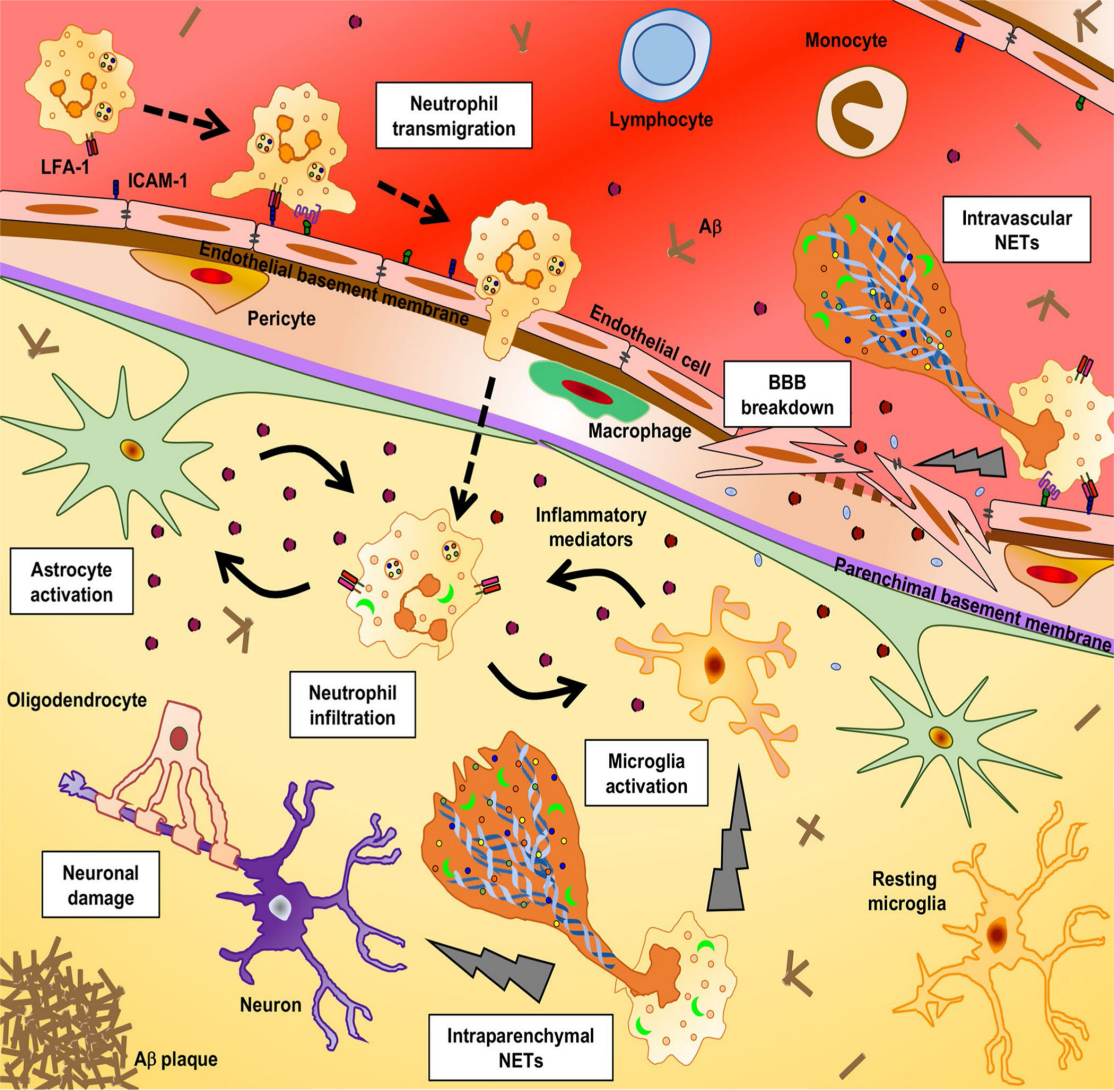
Schematic representation of BBB alterations and leukocyte extravasation in postcapillary venules in the AD brain. Endothelial cells are the first barrier between blood leukocytes and the brain parenchyma. Endothelial cells are linked by tight junctions closely surrounded by pericytes and encircled by the endothelial basal lamina (brown line) and parenchymal basement membrane (violet line). Astrocyte endfeet processes support endothelial functions and provide the cellular link to neuronal cells. Aβ and other inflammatory stimuli promote the activation of vascular endothelium, potentially inducing the expression of adhesion molecules and chemoattractants. The activated endothelium promotes the adhesion of neutrophils and eventually other circulating leukocytes that transmigrate into the brain parenchyma. Neutrophils adhered on the activated endothelium may release neutrophil extracellular traps (NETs) comprising decondensed chromatin and active proteases, which damage the BBB. Migrated neutrophils release inflammatory mediators and NETs, and may damage neurons. Neutrophils and glial cells may become trapped in a cycle of reciprocal activation, promoting chronic inflammation and neurodegeneration

Vascular inflammation in the context of an altered BBB has been implicated in the pathogenesis of AD [92, 93, 143, 144] (Fig. 5). Brain endothelial inflammation leads to the expression of two main classes of adhesion molecules: endothelial selectins such as E-selectin and P-selectin, and integrin ligands such as ICAM-1 and VCAM-1 from the Ig superfamily, which bind the LFA-1 and very late antigen 4 (VLA-4) integrins, respectively. During brain inflammation, ECs express adhesion molecules, which are also released in the bloodstream, providing biomarkers of vascular inflammation and BBB dysfunction. Soluble VCAM-1, ICAM-1, E-selectin and P-selectin were expressed at higher levels in plasma samples from AD patients compared to controls, suggesting that brain ECs are inflamed in AD [143]. For example, elevated levels of soluble VCAM-1 in the plasma of AD patients correlate with the severity of dementia and brain changes observed by magnetic resonance imaging (MRI) [143]. Interestingly, soluble adhesion molecules are also serum markers of inflammation and endothelial dysfunction associated with ageing, suggesting they may also represent a marker of age-dependent cognitive decline. Therefore, increased levels of soluble endothelial adhesion molecules may provide biomarkers of vascular inflammation and disease severity in AD.

Aβ peptides may directly activate ECs, inducing the expression of ICAM-1 and VCAM-1 and endothelial selectins, suggesting these molecules may promote leukocyte adhesion and transmigration during AD (Fig. 5). Accordingly, the expression of E-selectin, P-selectin, VCAM-1 and ICAM-1 is significantly higher in the brain vessels of transgenic animals with both Aβ and tau pathology compared to wild-type controls [144]. These adhesion molecules were expressed mainly in the brain vessels of the cortex, hippocampus, amygdala, meninges and choroid plexi of the AD mice [144]. However, it is unclear whether endothelial adhesion molecules are expressed at the BBB level in AD patients and further neuropathological studies are needed to clarify this key aspect of BBB dysfunction and brain inflammation.

Circulating leukocyte subpopulations, including monocytes, neutrophils and lymphocytes, have been identified in the brains of patients with AD and in transgenic animals with AD-like disease. Blood monocytes can migrate through the BBB into the brains of mice with AD-like disease using chemokine receptor CCR2 [87]. CCL2, the main CCR2 ligand, is expressed in the microvessels of AD-like mouse brains and the post-mortem brains of AD patients, suggesting it may also play a role in monocyte recruitment during human AD [87]. Several reports have indicated that migrating monocytes are beneficial in AD by promoting the clearance of Aβ (see [87]). However, more recent studies have challenged this view by showing that monocytes do not reduce the amyloid load, arguing against a role for circulating monocytes in Aβ clearance [97]. It is not yet known whether monocyte recruitment into the brain plays a role during the pathogenesis of human AD. Neutrophils can also migrate in the brains of AD patients and transgenic animals with AD-like disease [92, 93, 143, 144] (Fig. 5). These highly reactive cells do not need to accumulate in large numbers to induce substantial tissue damage, and may release a myriad of pro-inflammatory mediators including reactive oxygen species, enzymes and cytokines. Neutrophils may also release neutrophil extracellular traps (NETs), which can cause substantial tissue damage during inflammatory diseases [92, 93, 143, 144]. Recent studies have shown that neutrophils play a role in the induction of cognitive deficit and neuropathological changes in transgenic mice developing Aβ and tau pathologies [92, 93, 143, 144]. Neutrophils adhere inside blood vessels and migrate into the parenchyma in rodent AD models and human AD patients, whereas in controls these cells do not gain access to the CNS (Fig. 5). Intravascular and intra-parenchymal neutrophils also produce NETs in rodent AD models and human AD patients, suggesting these cells may induce BBB damage and harm neural cells. Interestingly, neutrophils adhere and spread inside blood vessels with Aβ deposits and migrate into the parenchyma in areas rich of with Aβ, supporting a role for Aβ in neutrophil recruitment in the AD brain (Fig. 5) [92, 93, 143, 144]. Soluble oligomeric Aβ1–42 induces the rapid, integrin-dependent adhesion of both human and mouse neutrophils on integrin ligands, triggering the LFA-1 integrin high-affinity state. These data suggest that vascular Aβ may favour neutrophil adhesion on brain ECs and may promote neutrophil-dependent BBB damage (Fig. 5). Therapeutic blocking of LFA-1 integrin rescues memory and reduces the severity of neuropathological changes in AD models, suggesting that interfering with the molecular mechanisms controlling leukocyte adhesion and migration inside the brain parenchyma may offer a new therapeutic strategy in AD.

CD4^+^ and CD8^+^ T cells can also adhere in brain vessels and migrate into the parenchyma in AD patients [143]. In agreement with these observations, patients with mild AD or MCI have greater numbers of activated CD4^+^ and CD8^+^ T cells in the CSF compared to controls, suggesting that lymphocytes with an activated phenotype can cross CNS barriers and contribute to disease pathogenesis [75]. In mouse models of Aβ pathology, T cells also infiltrate the brain and secrete interferon gamma (IFNγ) or IL-17, suggesting that lymphocyte cytokines may activate microglial cells and directly harm the BBB and neurons, promoting AD neuropathology. Peripheral inflammatory processes may activate T cells, promoting their translocation from the blood compartment into the brain, which may in turn contribute to AD pathology. In transgenic rodent models of Aβ pathology, the lack of lymphocytes reduces Aβ deposition enhances microglial activation and the phagocytosis of Aβ aggregates, suggesting that lymphocyte recruitment into the CNS may play a negative role in AD [118]. It remains unclear how T cells gain access through the BBB or other CNS barriers into the AD brain. Previous studies suggest that Aβ deposits, transforming growth factor p (TGF-β) and the expression of vascular adhesion molecules and chemoattractants may play a role in lymphocyte migration into the AD brain. As suggested for neutrophils, Aβ deposits promote T cell accumulation in the brain, presumably via the activation of brain ECs and the induction of vascular adhesion molecule expression [40]. Aβ1–42 induces the release of cytokines by microglial cells, which in turn promotes the trans-endothelial migration of T cells in vitro, whereas T cells were found in leptomeningeal and cortical vessels associated with CAA, suggesting that Aβ favours T cell migration into the AD brain [16, 140]. Furthermore, the injection of Aβ into the hippocampus activates ECs and promotes T cell migration into the brain [70]. TGF-β1 supports T cell infiltration in the meninges and brain parenchyma in mice immunised with Aβ1–42, but the role of this molecule in the neuro-inflammation associated with AD is still unclear [143]. Peripheral T cells in AD patients overexpress MIP-1α [77]. This may bind to its receptor CCR5, which is highly expressed on brain ECs in aged AD patients, providing supporting evidence for T cell migration through endothelial TJs into the CNS [77, 82].

## Concluding remarks

Since the concept and the cellular identity of the BBB have been established about 100 and 50 years ago, respectively, there has been considerable progress in understanding the molecular composition and genetic as well as to some extent epigenetic regulation. Specifically, the identification of pathways like Wnt/β-catenin, Shh, retinoic acid, angiopoietins and others, involved in regulation of barrier properties during embryonic and postnatal development as well as barrier maintenance, has provided novel insight into BBB characteristics. Moreover, these pathways offer novel strategies to mimic BBB function in single- and co-culture in vitro models and, none the less, novel targets for pharmacological opening or closing of the BBB under pathological conditions.

Nevertheless, many aspects of BBB dysfunction after stroke, in brain tumours, chronic inflammation and Alzheimer’s disease remain unknown. Future studies should aim to further elucidate the causes and consequences of BBB disruption, particularly with regard to the close interaction between neuronal, glial, PCs and ECs—i.e. the NVU—as well as inflammatory cells. In general, acute and chronic inflammation appears to be a hallmark of CNS pathology, requiring a re-adjustment of defining the concept that the CNS is an immune-privileged organ. In fact, targeting the immune response directly or indirectly by small-molecules or antibodies that cross the BBB may have beneficial effects by preventing for example cancer oedema, favouring Aβ clearance and decreasing chronic neuro-inflammation. In this context, the characterization of adhesion molecules and chemoattractants controlling vascular inflammation, as well as pathway that might be exploited to seal the BBB such as Wnt/β-catenin, Shh, retinoic acid, angiopoietins, etc. will be a highly relevant task in the future. Hence, combining multiparametric imaging information, (causal) relationships between BBB dysfunction and other (patho)biological factors, will enable original translational studies in experimental and clinical settings that will increase our understanding of the critical role of the BBB in brain pathology and recovery, ultimately contributing to improved patient care.

In conclusion, understanding the healthy BBB with its cellular and molecular players will be a prerequisite for transferring the knowledge to targeted therapy of CNS pathologies. Studies of recent years have shown that the BBB and the NVU in particular, is even more complex and diverse in specific regions of the CNs with regard to cellular and molecular interactions. The characterization of molecular mechanisms leading to BBB dysfunction and of common and diverse aspects of BBB function in the CNS will help to specifically modulated barrier characteristics on demand for the adjunct treatment of fatal diseases like stroke, brain tumour, chronic inflammation and Alzheimer’s disease.
